# Clinical characteristics and predictors for recurrence in chronic nonbacterial osteomyelitis: a retrospective multicenter analysis

**DOI:** 10.55730/1300-0144.5675

**Published:** 2023-02-28

**Authors:** Kadir ULU, Rana İŞGÜDER, Şerife Gül KARADAĞ, Esra BAĞLAN, Gülşah KAVRUL KAYAALP, Gülçin OTAR YENER, Kübra ÖZTÜRK, Hafize Emine SÖNMEZ, Semanur ÖZDEL, Ferhat DEMİR, Balahan MAKAY, Şevket Erbil ÜNSAL, Betül SÖZERİ, Nuray AKTAY AYAZ, Mustafa ÇAKAN

**Affiliations:** 1 Department of Pediatric Rheumatology, Ümraniye Training and Research Hospital, University of Health Sciences, İstanbul, Turkiye; 2 Department of Pediatric Rheumatology, Faculty of Medicine, Dokuz Eylül University, İzmir, Turkiye; 3 Department of Pediatric Rheumatology, Faculty of Medicine, İstanbul University, İstanbul, Turkiye; 4 Department of Pediatric Rheumatology, Dr. Sami Ulus Maternity and Child Health and Diseases Research and Training Hospital, University of Health Sciences, Ankara, Turkiye; 5 Department of Pediatric Rheumatology, Medical Point Hospital, Gaziantep, Turkiye; 6 Department of Pediatric Rheumatology, Göztepe Prof. Dr. Süleyman Yalçın City Hospital, İstanbul Medeniyet University, İstanbul, Turkiye; 7 Department of Pediatric Rheumatology, Faculty of Medicine, Kocaeli University, Kocaeli, Turkiye; 8 Department of Pediatric Rheumatology, Acıbadem Hospital, İstanbul, Turkiye

**Keywords:** Chronic nonbacterial osteomyelitis, chronic recurrent multifocal osteomyelitis, autoinflammation, children

## Abstract

**Background/aim:**

Chronic nonbacterial osteomyelitis (CNO) is a rare disease of unknown etiology and most commonly occurs during childhood or adolescence. The purpose of this study is to collect data on the clinical features, outcomes, and management of the disease and to identify the factors affecting recurrence.

**Materials and methods:**

This is a retrospective multicenter cross-sectional study of pediatric patients diagnosed with CNO. A total of 87 patients with a diagnosis of CNO followed for at least 6 months in 8 pediatric rheumatology centers across the country between January 2010 and December 2021 were included in this study.

**Results:**

The study included 87 patients (38 girls, 49 boys; median age: 12.5 years). The median follow-up time was 20 months (IQR: 8.5–40). The median time of diagnostic delay was 9.9 months (IQR: 3–24). Arthralgia and bone pain were the most common presenting symptoms. Multifocal involvement was detected in 86.2% of the cases and a recurrent course was reported in one-third of those included in the study. The most commonly involved bones were the femur and tibia. Vertebrae and clavicles were affected in 19.5% and 20.6% of cases, respectively. The erythrocyte sedimentation rate (ESR) values of 60.9% of the patients were above 20 mm/h and the C-reactive protein values of 44.8% were above 5 mg/L. The remission rate was 13.3% in patients using nonsteroidal antiinflammatory drugs and 75.0% in those using biological drugs. Vertebral and mandibular involvement and high ESR values at the time of diagnosis were associated with recurrence.

**Conclusion:**

In this multicenter study, CNO with vertebral and mandibular involvement and high ESR at diagnosis were associated with recurrence.

## 1. Introduction

Chronic nonbacterial osteomyelitis (CNO) is an aseptic autoinflammatory bone disorder of unknown etiology. CNO indicates bone inflammation regardless of the number of lesions; its more severe, recurrent, and multifocal form is called chronic recurrent multifocal osteomyelitis [1]. The classic sites of CNO involvement are the long bones of the lower extremities, clavicle, vertebrae, mandible, and pelvis. Although physical examination is usually normal, tenderness, swelling, or warmth may be present over the affected bones. Along with CNO, other inflammatory conditions affecting various organs can be observed, including severe acne, pyoderma gangrenosum, palmoplantar pustulosis, inflammatory bowel disease, and spondylarthritis [2]. Imaging studies, especially magnetic resonance imaging (MRI), are crucial in diagnosing and monitoring CNO and determining the numbers and sites of affected bones and responses to treatment [3].

The molecular basis of inflammation is associated with increased expression of inflammatory cytokines, namely interleukin (IL)-1β, IL-6, and tumor necrosis factor alpha (TNF-α), and decreased expression of immunomodulatory cytokines (i.e., IL-10 and IL-19). The resulting imbalance contributes to chronic tissue inflammation, osteoclast activation, bone resorption, and, in some cases, hyperostosis and bone sclerosis. Inflammation may affect all parts of the skeleton except the neurocranium [4]. First-line treatment usually includes nonsteroidal antiinflammatory drugs (NSAIDs) and/or glucocorticoids. Where these treatments are ineffective, a variety of medications are administered, including bisphosphonates, sulfasalazine, methotrexate, and anti-TNF agents [5].

CNO is a chronic disease with clinically active or inactive phases that may require long-term treatment. The disease has a high recurrence rate in long-term follow-up. The reported recurrence rate of this disease ranges widely from 16% to 83% [6]. In a study by Schnabel et al., at least one relapse was observed in 33 of 56 patients (58.9%) during the follow-up period [7]. In a study conducted in North America, a unifocal recurrent pattern was found in 25% of the patients and a multifocal recurrent pattern in 58% [8]. In the present study, we aimed to evaluate the demographics, clinical features, treatment modalities, outcomes, and factors affecting recurrence in children with CNO.

## 2. Materials and methods

A total of 87 patients diagnosed with CNO between January 2010 and December 2021 in 8 pediatric rheumatology centers across the country were enrolled in this multicenter, retrospective study. The patients were diagnosed based on the Bristol diagnostic criteria, according to which the diagnosis of CNO was made based on the presence of typical clinical findings (bone pain ± localized swelling without significant local or systemic features of inflammation or infection) and the presence of typical radiological findings (plain X-ray showing a combination of lytic areas, sclerosis, and new bone formation or preferably STIR MRI showing bone marrow edema ± bone expansion, lytic areas, and periosteal reaction). Patients with multiple bone involvement or clavicle involvement alone without significant elevation of C-reactive protein (CRP) above 30 g/L were diagnosed with clinical and imaging findings. Those with unifocal involvement (except clavicle) or those with CRP of >30 g/L were diagnosed with bone biopsy showing inflammatory changes (plasma cells, osteoclasts, fibrosis, or sclerosis) without antibiotic treatment and without bacterial growth [9]. Data on age at the onset of symptoms, age at diagnosis, gender, clinical features, CNO lesion distribution, laboratory features, comorbidities, medications, number of flares, and complications were noted using a standard form.

Pain and swelling, elevated inflammatory markers, and abnormal signals on MRI were defined as indicators of active disease. Clinically inactive disease/remission was defined as the resolution of the symptoms, normalization of inflammatory parameters, and radiographic improvement. Relapse was defined as the new onset of clinical symptoms after remission and/or the presence of new lesions on MRI [7,8].

The study was approved by the Local Ethics Committee of Ümraniye Training and Research Hospital (17.09.2020, B.10.1.TKH.4.34.H.GP.0.01/309) and was performed according to the tenets of the Declaration of Helsinki. Written informed consent was received from the legal guardians of all patients.

### 2.1. Statistical analysis

Analyses were performed using IBM SPSS Statistics 25.0 (IBM Corp., Armonk, NY, USA). The normality of numerical variables was determined by Kolmogorov–Smirnov test. Descriptive statistics were reported as mean ± standard deviation (SD) or median [interquartile range (IQR)] and percentage values. Intergroup comparisons were performed with Fisher’s exact or chi-square tests as appropriate. The Mann–Whitney U test was applied to compare numerical variables between groups. The variables were tested in regression analysis for the assessment of risk factors for relapse. The variance inflation factor was used to reduce multicollinearity. The level of statistical significance was defined as a 2-tailed p-value of <0.05.

## 3. Results

Eighty-seven patients (56.3% male) were assessed with a median follow-up of 20 months (IQR: 8–40). The mean age at diagnosis was 12.2 ± 4.4 years. The median time of diagnostic delay was 9.9 months (IQR: 3–24). Before the diagnosis, cast immobilization was applied for 9 patients and surgical intervention for 3 patients. Arthralgia in various joints, especially the ankle joints, and bone pain were the most common presenting symptoms (93.1%). Multifocal involvement was observed in the majority of patients during the follow-up period and recurrence was observed in 29 (33.3%) patients. The number of flares per patient varied between 1 and 5.

**Figure 1:**
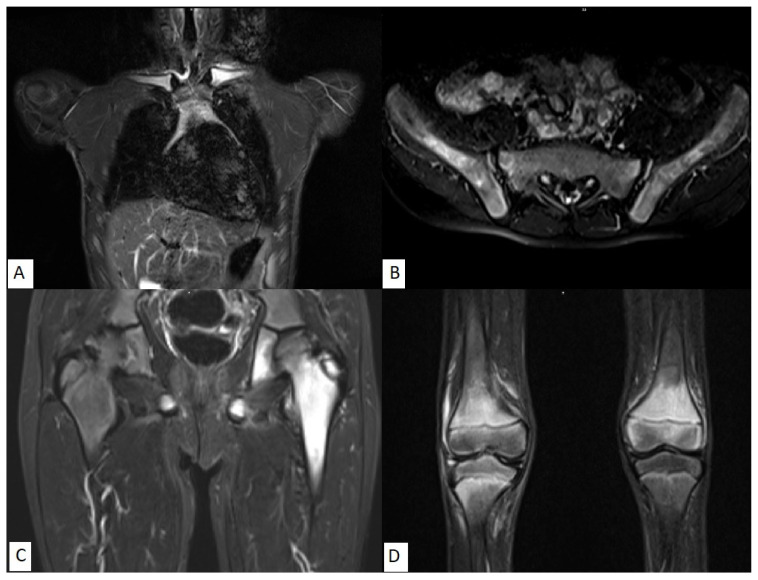
Magnetic resonance imaging of chronic nonbacterial osteomyelitis patients. Hyperintense signal changes in T2-weighted sequences are observed. **A)** Imaging results of a 17-year-old male patient, in the metaphyseal part of the left clavicle and adjacent soft tissue; **B**) a 12-year-old female patient, in the right ilium; **C)** a 13-year-old male patient, in the ischium, greater trochanter, and metaphysis of the left femur; **D)** a 12-year-old female patient, in the distal epiphysis of the right femur, in the proximal metaphysis of the right tibia, and in the distal epiphysis of the left femur.

The distribution of the lesions was examined by MRI (local MRI for 57.5% and whole-body MRI for 42.5% of the patients) at the time of diagnosis and throughout the disease course. The metaphyses of the long bones, and especially the lower extremities (femur, tibia, and metatarsal bones), were affected most frequently (77%). The most commonly involved bones were the femur (n = 38), tibia (n = 38), and ilium (n = 25). Throughout the disease course, 273 lesions were detected, with a median of 3.1 lesions per child. Vertebrae and clavicles were affected in 17 (19.5%) and 18 (20.6%) cases, respectively. MRI images of commonly involved bones such as the left clavicle (Figure 1A), right ilium (Figure 1B), left femur (Figure 1C), and bilateral femurs and right tibia (Figure 1D) are presented in Figure 1.

Peripheral arthritis was found in 52 patients (59.7%), isolated sacroiliitis in 15 patients (17.2%), and both types of involvement in 5 patients (7.4%). The most frequently involved joints were found to be foot and ankle joints. In 48 cases, the arthritis was adjacent to the affected bone, while in 4 cases it was distant.

Extensive acne was present in 2 cases (2.3%), psoriasis in 2 cases (2.3%), and inflammatory bowel disease in 1 case (1.1%) as comorbid disorders.

The median erythrocyte sedimentation rate (ESR) value at the time of diagnosis was 32 (IQR: 13–50) mm/h. The ESR value of 53 (60.9%) patients was above 20 mm/h. The median CRP value at the time of diagnosis was 6.9 (IQR: 1.5–30.4) mg/L. The CRP value of 39 (44.8%) patients was above 5 mg/L. Biopsy was performed for 36 patients (41.3%) with unifocal lesions, atypical involvement, or suspected malignancy. The clinical features, laboratory features, sites of involvement, and imaging methods of the patients are summarized in the Table.

NSAIDs and glucocorticoids were used as first-line therapy. Disease-modifying antirheumatic drugs (DMARDs), anti-TNF agents, and bisphosphonates were used as second-line therapy. Etanercept was started for 12 patients and adalimumab was started for 16 patients as the first biological agent. Compared to those with unifocal nonrecurrent involvement, there was a statistically significant increase in the use of biologics in other clinical forms (p = 0.001). NSAIDs were used for 83 of the 87 (95.4%) patients included in the study and inactive disease was achieved in 11 (13.3%) cases. Glucocorticoid was used as bridge therapy for 40 (45.9%) patients. DMARDs were used for 76 (87.3%) patients and inactive disease was achieved in 40 (52.6%) cases. Anti-TNF agents were used for 28 of 34 patients with recurrent and resistant courses, while bisphosphonates were used for 6 of them. Inactive disease was achieved in 21 (75%) of those using anti-TNF agents. Three of 6 patients using bisphosphonates reached an inactive disease state.

Recurrence was observed in 29 (33.3%) cases. The mean recurrence time after remission was 9.9 ± 7.8 months. Patients with relapse had significantly elevated ESR values at the time of diagnosis [43 mm/h (IQR: 24.5–70.2) versus 24 mm/h (IQR: 10.5–43.5), p = 0.007].

In regression analysis, the presence of relapse was independently associated with vertebral involvement (p = 0.03, 95% CI: 0.022–0.521) and mandibular involvement (p = 0.03, 95% CI: 0.236–1.139). No significant difference was found among patients with relapse in terms of age, age at diagnosis, gender, or diagnosis delay time. While the same bone region involvement was observed in 19 (21.8%) patients with recurrence, new bone region involvement was observed in 10 (11.4%) patients.

At the last examination, the disease was active in 14 (16.1%) cases while 73 (83.9%) patients had achieved complete remission. Complications developed in only 9 (10.3%) cases, including fractures in 6 cases and osteoporosis in 3 cases.

## 4. Discussion

This large multicenter cohort provides a detailed assessment of the clinical findings, the extent of involvement, therapeutic approaches, and factors influencing relapse in children with CNO. It can be said that the findings reflect the characteristics of CNO on a nationwide basis since patients followed in 8 pediatric rheumatology centers from different cities of Türkiye were included. We found that the metaphyses of the lower-extremity long bones were the most frequently affected regions in CNO. Most patients had multifocal involvement and one-third had a recurrent course. Vertebral and mandibular involvement and high ESR values at the time of diagnosis were associated with recurrence.

Girschick et al. [1] recently published the largest case series of CNO patients to date. They reported 486 patients, including 310 female patients and 30 adults. In the literature, CNO is generally more common in female patients [10,11]. In contrast, 53.6% of our cohort were male. Consistent with the current study, Sözeri et al. [12] found this rate to be 52.9% in a previous study from Türkiye. In another study conducted by Concha et al. [13], 53% of the included patients were male.

The mean age at diagnosis obtained in the present study was similar to that of previously reported research. However, the time elapsed from symptom onset to diagnosis differed among various cohorts from 3 months to 17 months [7–11,14]. Low awareness of the disease among physicians, nonspecific examination findings, lack of definitive diagnostic criteria, and the fact that CNO is still a diagnosis of exclusion may explain the delays in diagnosis. CNO should be considered in children with localized bone pain, and also in cases of osteomyelitis that do not respond to antibiotic treatments.

CNO remains a diagnosis of exclusion, requiring the exclusion of malignancy, infection, and other bone disorders. When a disease-specific region such as the clavicle is not involved, it is important to exclude bacterial osteomyelitis and other malignant disorders with biopsy, especially in patients with unifocal bone involvement.

The ESR and CRP values were moderately high in this study, comparable to other studies [7,10–15]. However, the ESR values of our patients with recurrence were significantly higher at admission compared to those with nonrecurrent courses. It is important to closely follow patients with high ESR values at the time of diagnosis to predict or prevent possible relapses.

Multifocal involvement is the most common form of bone lesions in CNO [1,15,16]. In the study conducted by Ma et al. [17], the median number of bones involved was 5. Girschick et al. [1] reported that the number of lesions per patient was 1.9 by X-ray, 3.5 by scintigraphy, and 4.1 by MRI. In the current study, all patients were evaluated with MRI and the mean number of affected bones was 3.1. In light of this information, it is clear that MRI is more sensitive in detecting silent lesions and is safer than scintigraphy because it does not entail radiation exposure.

Consistent with the literature, the most frequently involved bones in our cohort were those of the lower limbs [1,10–15]. Specifically, the most frequently involved bones were the femur and tibia. Previous studies reported spinal involvement in 20%–46% of CNO patients [18]. In our study, vertebral involvement was observed in 18.4% of the cases and the recurrence rate was significantly higher among patients with vertebral involvement. Spinal involvement is regarded as the most important involvement in CNO because it causes severe complications such as kyphosis, neurological dysfunction, and spinal cord compression. Therefore, it is recommended that all patients be screened for vertebral involvement at the time of diagnosis, even if they have no complaints [3]. A multidisciplinary approach and aggressive treatment plan seem to be reasonable options in cases of vertebral CNO.

In CNO, mandibular involvement is less commonly observed than other bone involvements [11,12]. In one previous study, 22 CNO patients with mandibular involvement were evaluated and anti-TNFs and bisphosphonates were found more effective than first-line treatments [19]. Although mandibular involvement was less common in our cohort, the recurrence rate was significantly higher compared to other bone involvements. To prevent complications and reduce morbidity, more aggressive treatment is important in cases of mandibular involvement.

NSAIDs are considered first-line therapy in the treatment of CNO. Various treatment options including methotrexate, sulfasalazine, biologic drugs, and bisphosphonates have been used for patients who do not respond to NSAIDs or have recurrences [16,20,21]. NSAIDs appear to be partially effective in controlling the disease in a minority of the patients. Glucocorticoids can be used as bridging therapy, but they should be administered with caution due to their long-term side effects. Recently, Schnabel et al. [20] reported that more than 60% of NSAID-treated patients experienced exacerbations, while 39 of 43 (90.6%) anti-TNF-treated patients achieved partial or complete remission for 12 months. These results are comparable to ours. In the same study, 35 of 51 (68.6%) patients treated with pamidronate reached complete remission within 12 months. In our cohort, anti-TNFs were more commonly preferred. Bisphosphonates have effects on osteoclasts and inhibit proinflammatory cytokines. Many studies have shown their effectiveness in CNO [5,21]. In another study, while the response to pamidronate was good in the first year, failure and recurrences were observed afterwards, and it was emphasized that a biological agent may be required in the treatment of active CNO and synovitis [22].

One limitation of this study is that clinical data and imaging results were obtained from medical records with a retrospective research design. Furthermore, treatment responses for different regimens could not be adequately compared due to the low number of patients using bisphosphonates. Since this disease is extremely rare, it is hard to create CNO cohorts with large numbers of patients. The main strength of the study was that it was a multicentric nationwide study with a relatively large number of CNO patients.

In conclusion, CNO patients with vertebral and mandibular involvement and those with high ESR values at the time of diagnosis should be followed carefully in terms of recurrence. NSAIDs alone were insufficient to control disease activity in most cases, but disease activity was controlled by the addition of methotrexate/salazopyrin or a biological agent for most patients.

## Figures and Tables

**Table:** Clinical and laboratory findings of the CNO patients.

Disease course, n (%)	
Unifocal and nonrecurrent	8 (9.2)
Unifocal and recurrent	4 (4.6)
Multifocal and nonrecurrent	50 (57.5)
Multifocal and recurrent (CRMO)	25 (28.7)
Clinical presentation, n (%)	
Bone pain	70 (80.4)
Arthralgia	48 (43.6)
Swelling	20 (22.9)
Limp	23 (26.4)
Bone involvement, n (%)	
Femur	38 (43.7)
Tibia	38 (43.7)
Pelvic bones	36 (41.3)
MTP	21 (24.1)
Clavicle	17 (19.5)
Tarsal bones	17 (19.5)
Vertebra	16 (18.4)
Humerus	9 (10.3)
Sternum	2 (2.3)
MCP	6 (6.9)
Ulna	5 (5.7)
Radius	4 (4.6)
Fibula	5 (5.7)
Scapula	3 (3.4)
Rib	3 (3.4)
Mandible	4 (4.6)
Carpal bones	1 (1.1)
Laboratory investigations (baseline)	
HLA-B27 positivity, n (%)	9/70 (12.8)
ANA positivity, n (%)	13/76 (17.1)
CRP, mg/L, mean (SD)	20.6 (31.3)
ESR, mm/h, mean (SD)	37.4 (30.8)
Imaging methods, n (%)	
Whole body STIR MRI	37 (42.5)
Local MRI	50 (57.5)

CNO: Chronic nonbacterial osteomyelitis; MTP: metatarsal phalanx; MCP: metacarpal phalanx; STIR MRI: short tau inversion recovery magnetic resonance imaging; HLA: human leukocyte antigen; ANA: antinuclear antibody; CRP: C-reactive protein.

## Data Availability

The datasets used in this study are available from the corresponding author on reasonable request.

## References

[1] Girschick H, Finetti M, Orlando F, Schalm S, Insalaco A (2018). The multifaceted presentation of chronic recurrent multifocal osteomyelitis: a series of 486 cases from the Eurofever international registry. Rheumatology (Oxford).

[2] Nuruzzaman F, Zhao Y, Ferguson PJ (2021). Chronic nonbacterial osteomyelitis: insights into pathogenesis, assessment, and treatment. Rheumatic Diseases Clinics of North America.

[3] Zhao Y, Wu EY, Oliver MS, Cooper AM, Basiaga ML (2018). Consensus treatment plans for chronic nonbacterial osteomyelitis refractory to nonsteroidal antiinflammatory drugs and/or with active spinal lesions. Arthritis Care & Research.

[4] Hedrich CM, Morbach H, Reiser C, Girschick HJ (2020). New insights into adult and paediatric chronic non-bacterial osteomyelitis CNO. Current Rheumatology Reports.

[5] Ramanan AV, Hampson LV, Lythgoe H, Jones AP, Hardwick B (2019). Defining consensus opinion to develop randomised controlled trials in rare diseases using Bayesian design: an example of a proposed trial of adalimumab versus pamidronate for children with CNO/CRMO. PLoS One.

[6] Zhao DY, McCann L, Hahn G, Hedrich CM (2021). Chronic nonbacterial osteomyelitis (CNO) and chronic recurrent multifocal osteomyelitis (CRMO). Journal of Translational Autoimmunity.

[7] Schnabel A, Range U, Hahn G, Berner R, Hedrich CM (2017). Treatment response and longterm outcomes in children with chronic nonbacterial osteomyelitis. Journal of Rheumatology.

[8] Borzutzky A, Stern S, Reiff A, Steinberg EA, Dedeoglu F (2012). Pediatric chronic nonbacterial osteomyelitis. Pediatrics.

[9] Roderick MR, Shah R, Rogers V, Finn A, Ramanan AV (2016). Chronic recurrent multifocal osteomyelitis (CRMO) - advancing the diagnosis. Pediatric Rheumatology Online Journal.

[10] d’Angelo P, de Horatio LT, Toma P, Ording Müller LS, Avenarius D (2021). Chronic nonbacterial osteomyelitis - clinical and magnetic resonance imaging features. Pediatric Radiology.

[11] Wipff J, Costantino F, Lemelle I, Pajot C, Duquesne A (2015). A large national cohort of French patients with chronic recurrent multifocal osteitis. Arthritis & Rheumatology.

[12] Sözeri B, Ayaz NA, Atıkan BY, Karadağ ŞG, Çakan M (2019). Clinical experiences in Turkish paediatric patients with chronic recurrent multifocal osteomyelitis. Turkish Journal of Pediatrics.

[13] Concha S, Hernández-Ojeda A, Contreras O, Mendez C, Talesnik E (2020). Chronic nonbacterial osteomyelitis in children: a multicenter case series. Rheumatology International.

[14] Okay E, Ulu K, Demir F, Sari T, Zeynalov S (2023). Chronic recurrent multifocal osteomyelitis: a multidisciplinary experience of 22 pediatric cases with a mean follow-up of 27 months. Journal of Orthopaedic Science.

[15] Bhat CS, Anderson C, Harbinson A, McCann LJ, Roderick M (2018). Chronic nonbacterial osteitis- a multicentre study. Pediatric Rheumatology Online Journal.

[16] Açarı C, Çomak E, Çekiç Ş, Türkuçar S, Adıgüzel Dündar H (2021). Clinical features of children with chronic non-bacterial osteomyelitis: a multicenter retrospective case series from Turkey. Archives of Rheumatology.

[17] Ma L, Liu H, Tang H, Zhang Z, Zou L (2022). Clinical characteristics and outcomes of chronic nonbacterial osteomyelitis in children: a multicenter case series. Pediatric Rheumatology Online Journal.

[18] Kostik MM, Kopchak OL, Maletin AS, Mushkin AY (2020). The peculiarities and treatment outcomes of the spinal form of chronic non-bacterial osteomyelitis in children: a retrospective cohort study. Rheumatology International.

[19] Gaal A, Basiaga ML, Zhao Y, Egbert M (2020). Pediatric chronic nonbacterial osteomyelitis of the mandible: Seattle Children’s Hospital 22-patient experience. Pediatric Rheumatology Online Journal.

[20] Schnabel A, Nashawi M, Anderson C, Felsenstein S, Lamoudi M (2022). TNF-inhibitors or bisphosphonates in chronic nonbacterial osteomyelitis? - Results of an international retrospective multicenter study. Clinical Immunology.

[21] Timme M, Bohner L, Huss S, Kleinheinz J, Hanisch M (2020). Response of different treatment protocols to treat chronic non-bacterial osteomyelitis (CNO) of the mandible in adult patients: a systematic review. International Journal of Environmental Research and Public Health.

[22] Andreasen CM, Jurik AG, Glerup MB, Høst C, Mahler BT (2019). Response to early-onset pamidronate treatment in chronic nonbacterial osteomyelitis: a retrospective single-center study. Journal of Rheumatology.

